# Rhizosphere legacy of leaf-diseased rice and its impact on next generation

**DOI:** 10.3389/fmicb.2025.1677271

**Published:** 2025-12-17

**Authors:** Léa Jobert, Vichny Vicheth, Pierre Czernic, Malyna Suong, Gilles Béna, Lionel Moulin

**Affiliations:** 1 Plant Health Institute of Montpellier (PHIM), IRD, CIRAD, INRAE, Institut Agro, Univ Montpellier, Montpellier, France; 2 Research and Innovation Center, Institute of Technology of Cambodia, Phnom Penh, Cambodia

**Keywords:** disease-induced microbiome, dysbiosis, *Oryza sativa*, plant microbiome, plant-soil feedback

## Abstract

Plants interact continuously with the surrounding soil microbiota, shaping and being shaped by these communities over time, termed as the plant-soil feedback (PSF). As a result, plants can leave a biological imprint in the soil that affects the performance of subsequent plants, a phenomenon termed the soil-borne legacy. In this study, we investigated how the rhizosphere microbiota of rice plants exhibiting (or not) foliar disease symptoms in a Cambodian field was modified and influenced subsequent generation of plants. Based on a visual assessment of foliar symptoms, we collected and mixed the rhizospheres of plants classified as “diseased” or “healthy,” respectively. These mixed rhizospheres were then used to sow new rice plants in controlled conditions, which were subsequently challenged with *Xanthomonas oryzae* pv. *oryzae*. Phenotypic analyses revealed that plants grown in the rhizosphere microbiota collected from diseased field plants were smaller, yet displayed smaller symptoms to foliar pathogens compared to those grown in the microbiota of healthy plants. Amplicon sequencing of roots and rhizospheres from field samples confirmed that diseased and healthy plants harbored distinct microbial communities. A dysbiotic rhizosphere was found to be present in leaf-diseased plants, in contrast to healthy ones. These differences were also detectable in the composite rhizosphere mixes, and persisted in the rhizospheres of a new generation of rice plants grown in these soils. This suggests a microbiota-driven legacy, wherein the health status of the previous generation shapes the microbial environment and influences plant phenotype in terms of growth and defense. Our results support the idea that leaf-diseased plants condition their rhizosphere microbiota thus influencing plant phenotype in the next generation. Understanding the impact of disease-induced microbial legacy on next generation plant phenotype is crucial for developing microbiome-based crop protection strategies.

## 1 Introduction

The plant-associated microbiome plays a vital role in plant health by modulating growth, improving nutrient acquisition, and priming immune responses (Berendsen et al., 2012; Pieterse et al., 2014). Under certain conditions, it can also suppress soil-borne diseases (Weller et al., 2002). Plants interact continuously with the surrounding soil microbiota, shaping and being shaped by these communities over time, termed as the plant-soil feedback (PSF). As a result, plants can leave a biological imprint in the soil that affects the performance of subsequent plants, a phenomenon termed the soil-borne legacy. PSF encompasses both biotic and abiotic modifications to the soil environment driven by plant activity (Bakker et al., 2018). One biotic mechanism underlying a positive soil-borne legacy is the accumulation of beneficial microbes, which can increase disease resistance or nutrient efficiency in the next generation (Yuan et al., 2018; Backer et al., 2018). In response to pathogen attack, plants can modify the composition of their root exudates to recruit beneficial microbes from the soil (Baetz and Martinoia, 2014). This hypothesis, referred to as the “cry for help’’, suggests that pathogen pressure can actively shape protective microbial communities in the rhizosphere (Rolfe et al., 2019). According to this hypothesis, targeted exudates recruit plant growth-promoting microbes (PGPMs) that assemble into a protective consortium capable of priming systemic immunity (Pieterse et al., 2014). If successful, this recruitment may not only help the infected plant but also leave behind a microbial legacy that benefits the next generation. Over time, such beneficial feedbacks may contribute to the development of disease-suppressive soils - soils in which plant pathogens are present, but disease levels remain low due to microbial antagonism or induced resistance mechanisms (Berendsen et al., 2012; Mendes et al., 2011).

Take-all decline is a classic example of disease suppression (Weller et al., 2002), but the literature continually adds to the list of examples, including pathogens from different kingdoms such as *Rhizoctonia solani*, *Fusarium oxysporum*, *Meloidogyne* spp. or *Ralstonia solanacearum* (Spooren et al., 2024; Mendes et al., 2011). Importantly, PSF-driven suppressiveness is not restricted to root diseases; conditioning the soil microbiome can also reduce attacks by above-ground herbivores and foliar pathogens (Pineda et al., 2017; Yi et al., 2011). Nevertheless, below-ground recruitment mediated by foliar pathogens remains sparsely documented. Studies on this subject tend to focus on the *Arabidopsis thaliana* pathosystem, which is challenged by the biotrophic downy mildew pathogen *Hyaloperonospora arabidopsidis* (*Hpa*). Following *Hpa* infection, the roots specifically attracted *Bacillus*, *Microbacterium* and *Pseudomonas* strains that suppress disease (Berendsen et al., 2018). Another study reported that plants impaired in defense signaling via the plant hormone salicylic acid are not protected by an *Hpa*-induced soil-borne legacy (Vismans et al., 2022). This suggests that the legacy may enable defense by affecting salicylic acid-dependent immunity in plants. Recent work on *Arabidopsis* infected by the foliar pathogen *Pseudomonas syringae* DC300 (*Pst*) has shown that repeated selection by a foliar pathogen can leave a plant-soil feedback that enhances resistance in the next generation (Kalachova et al., 2022).

Conversely, persistent imbalances of the microbiome, or dysbiosis, can disrupt plant-microbe beneficial interactions, increasing plant susceptibility. Dysbiosis is defined as a transitory loss of the host’s capacity to regulate its microbiota, implying a loss of function that leads to a reduction in the host’s fitness (Arnault et al., 2023). It may result from biotic (e.g., pathogens or herbivores) or abiotic (e.g., drought or fertilization) stressors affecting the host, the microbiota or both. Furthermore, dysbiosis can be a cause or a consequence of disease development (Arnault et al., 2023). Indeed, it has been shown that a pathogen disrupts the rhizosphere microbiome during an invasion as it is for example the case with the tomato rhizosphere microbiome following *Ralstonia solanacearum* invasion (Wei et al., 2018). On the other hand, variations in initial soil microbiome composition can predetermine future plant health indicating that the spatial heterogeneity of microbial communities in the field plays a critical role in shaping plant health trajectories (Wei et al., 2019; Gu et al., 2022). Unfortunately, few studies have examined the impact of foliar disease on the microbiota associated with roots in the field, or its effect on subsequent generations. Understanding how differences in soil microbiota affect disease outcomes is particularly relevant for major crops like rice, especially in regions where pathogen pressure is high.

Rice (*Oryza sativa*) is a staple crop for millions of people in Southeast Asia, including in Cambodia, where it is fundamental to food security and rural livelihoods. However, around 30% of plant yield is lost in Southeast Asia every year due to pathogen infections (Savary et al., 2019). Bacterial Leaf Blight (BLB) caused by *Xanthomonas oryzae* pv. *oryzae* is the most impacting bacterial disease worldwide, leading up to 50% yield loss in severely infested fields (Lee et al., 2005; Savary et al., 2019). While some studies have focused on rice microbiome assembly (Edwards et al., 2015), very few have focused on a microbiota assembly driven by disease in this same crop. Recently, Jiang et al. analyzed the impact of rice bacterial leaf blight (BLB) on rhizosphere microbial assembly (Jiang et al., 2023). They discovered that communities of rhizosphere bacteria were more sensitive to BLB leaf infection than fungal communities, and that the abundance of certain genera such as *Streptomyces, Chitinophaga, Sphingomonas,* and *Bacillus*, increased significantly in infected plants. No significant modification of the bacterial and fungal microbiome diversities was found due to blast disease on rice plants from the field, but the community structure in the root and rhizosphere compartments was altered (Dastogeer et al., 2022). Recent work also described health and disease taxa signature in the leaf, root and rhizosphere of rice, when comparing samples from healthy and diseased rice (Oeum et al., 2024). It is crucial to understand the rhizosphere microbial dynamics associated with healthy versus diseased rice plants, and the legacy effects of these dynamics on subsequent generations, in order to develop microbiome-based disease management strategies adapted to rice systems.

Here, we explored whether the health status of rice plants in the field leaves behind a microbial “legacy” that modulates the growth and resistance of the next generation of plants. To investigate this, (i) symptomatic and asymptomatic plants were collected from a Cambodian rice field, (ii) The rhizosphere of the two types of health status were mixed separately to form two soils labeled as diseased or healthy, (iii) a new generation of plants was sown in both rhizosphere mixtures and their growth and resistance phenotype analyzed to observe the effect of the rhizosphere origin on the growth and resistance of a new generation of plants. Microbiome sequencing was performed on the leaves, roots and rhizosphere compartment of field plants, as well as on the rhizosphere compartment of the next generation of plants grown under controlled conditions to analyze the modification of the microbiomes all along the steps of the study.

## 2 Materials and methods

### 2.1 Field sampling

Field sampling was conducted in January 2024 in the Kanghot perimeter, near Battambang, Cambodia (GPS coordinates: 12°54′40.2”N 103°16′26.1″E). The field was part of a large agroecology project (EU-AFD WAT4CAM project) comparing conservation and conventional agriculture in farmer fields. In the sampled field, two cycles of rice were performed in 2024, using the short cycle (3 months) local variety Srangae Sral (*Oryza sativa* subspecies *indica*). Rice was grown under conventional practice, with chemical fertilizers and pesticides inputs. Sampling was performed 2 months after sowing the second cycle, at the ripening stage. The leaves and root systems, along with their surrounding rhizosphere, were harvested based solely on foliar visual symptoms. Six symptomatic (“diseased”) plants and six asymptomatic (“healthy”) plants were individually collected from different locations in the field. For each plant, roots and rhizosphere samples were kept in sealed plastic bags, while leaves were stored individually in a paper notebook for 24 h before processing. Back in the laboratory, root systems and rhizosphere samples were separated. Roots of each plant were washed individually with autoclaved distilled water. Washed roots and leaves of each plant were grinded in liquid nitrogen using a sterile mortar, and 250 mg of the resulting powder was transferred into PowerBead tubes and stored at −80 °C for further DNA extraction. A small fraction of the rhizosphere (each being 5 sub-samples sampled randomly and mixed) was transferred into PowerBead tubes (Qiagen, Promega, United States) and stored at −80 °C until DNA extraction.

### 2.2 Plant growth system of subsequent plant generation under controlled conditions

Rhizosphere soil collected from the field was used for a new cycle of plant growth in controlled conditions. Large debris such as roots, insects and stones were first removed. The rhizospheres of the six non-symptomatic plants were pooled to form the “Healthy Rhizosphere Mix” (HRM), while the rhizospheres from the six symptomatic plants were combined to form the “Diseased Rhizosphere Mix” (DRM). Both rhizosphere mixtures were left to stabilize at room temperature for 3 weeks. The two mixes of rhizosphere (HRM/DRM) were diluted with perlite and vermiculite at ratio 20/50/30, then distributed into 450 mL pots. Seeds of the Srangae Sral rice variety—identical to those sampled in the field—were used. After 5 days of pregermination in sterile wet sand, three seedlings were transplanted per pot. Plants were maintained under a 12-h light/12-h dark photoperiod and watered with tap water.

### 2.3 Pathogen inoculation and plant phenotyping

After 5 weeks of growth, the size of the plants was measured by taking the length of the aerial parts from the collar to the tip of the longest leaf. On the same day, the plants were inoculated with *Xanthomonas oryzae* pv. *oryzae* (Xoo), the bacterium that causes bacterial leaf blight. Plants were inoculated by the leaf clipping method using a protocol adapted from Yuan et al. (2016). The Xoo strain CIX4551, previously isolated from Cambodian rice fields (Oeum et al., 2024), was cultured on PSA medium plates (10 g peptone, 10 g sucrose, 1 g glutamic acid, 16 g agar per liter distilled water) at 28 °C for 2 days, then bacterial colonies were recovered from plates using 1 mL of water, and the inoculum was prepared at an OD600 of 0.2 in sterile water supplemented with 0.025% Tween 80. Inoculation was performed by cutting the tip of the most recently fully developed leaf and dipping it into the bacterial suspension for 3 s. To confirm that symptom development was due to Xoo infection and not the inoculation method, a mock control pot was included, where plant leaves were dipped into the same solution but without bacteria. Disease symptoms were assessed 14 days post-inoculation by measuring the length of symptoms from the cutting site (Yuan et al., 2016).

For the phenotypic data analysis, biostatistics were performed in R version 4.5.0, using RStudio and RMarkdown version 2.29. The assumptions of normality and homogeneity of variances were assessed. When these assumptions were not met, non-parametric Wilcoxon rank-sum tests were applied using the wilcox.test() from *stats* (v4.5.0).

### 2.4 Plant harvesting, DNA extraction and amplicon-barcoding data production

Sampling of plants was performed 15 days post-inoculation, the day after symptom phenotyping. Plants were uprooted, and three plants from the same pot were pooled to form one sample as the roots were too tangled up together to be separated. Roots were placed in a ziplock bag and shaken to remove the attached rhizosphere, from which up to 250 mg were then randomly collected for further DNA extraction. Roots were subsequently washed with sterile water, ground in liquid nitrogen, and the resulting powder was used for DNA extraction. A negative control without a sample was included to monitor potential environmental and kit contaminants. DNA extraction was performed using the DNeasy PowerSoil Pro Kit (Qiagen, Promega, United States) according to the manufacturer’s protocol.

Quality control of DNA, PCR amplification, library construction, and Illumina MiSeq sequencing were carried out by Macrogen (Seoul, South Korea). The V3-V4 region of the 16S rRNA gene was amplified using the primers 341F (5′-CCTACGGGNGGCWGCAG-3′) and 805R (5′-GACTACHVGGGTATCTAATCC-3′) (Sinclair et al., 2015). For fungal communities, the ITS2 region of the rDNA Internal Transcribed Spacer (ITS) was amplified using the primers 3F (5′-GCATCGATGAAGAACGCAGC-3′) and 4R (5′-TCCTCCGCTTATTGATATGC-3′) (White et al., 1990). Libraries were sequenced on an Illumina MiSeq platform using a 2 × 300 bp paired-end approach. The number of reads obtained ranged from 35 k to 160 k per sample for 16S libraries, with an average of 53 k reads, and from 27 k to 124 k per sample for ITS libraries, with an average of 49 k reads. The raw sequencing data (FASTQ files) generated in this study have been deposited in the European Nucleotide Archive (ENA^1^) under the Bioproject accession PRJEB84191.

### 2.5 Bioinformatic analysis of 16S V3V4 and ITS2 amplicon sequences

Demultiplexed Fastq files of each marker per sample were analyzed using the DADA2 pipeline (Callahan et al., 2016) in R (version 4.5.0). Briefly, sequences were curated from primers using the trimleft option using the primer length of each primer, and cut with the truncLen() option at 280 bp (forward) and 205 bp (reverse) according to quality profiles of forward and reverse libraries, and filtered with maxN = 0 and maxEE = c(2,2). Then the learnErrors() function was applied (using the randomize = TRUE option) and errors were corrected using the dada() functions. Sequences were then merged into Amplicon Sequence Variant (ASV), and the taxonomy of obtained ASV was assessed using the assignTaxonomy function on Silva 138.2 (16S V3V4 sequences) (Quast et al., 2012) or Unite all eukaryotes (sh_general_release_dynamic_s_all_19.02.2025, ITS2) (Abarenkov et al., 2024). Taxonomic affiliations were refined by blast analyses on the top 100 taxa for both markers using the nr and 16S rRNA/ ITS database in NCBI.^2^ ASVs were filtered on their abundance (removing ASVs < 10 reads among all libraries), and ASVs present in the negative control were also filtered from the datasets. ASVs affiliated with chloroplasts or mitochondria, or belonging to the Viridiplantae kingdom, were removed from the 16S dataset. All ASVs that were not assigned to the Fungi or Metazoa kingdoms were also removed from the ITS dataset. ASVs that were not unassigned at the Kingdom or Phylum taxonomic level were also removed. Final datasets after quality filtering contained 11,498 ASVs for the 16S rRNA gene V3V4 amplicon and 1,658 ASVs for ITS2 amplicon.

All downstream analyses were performed in R version 4.5.0, using RStudio and RMarkdown version 2.29. Rarefaction curves were generated using ggrare() from *ranacapa* (v0.1.0) and visualized with *scales* (v1.4.0) and alpha diversity plots were generated using the *phyloseq* (v1.53.0) (McMurdie and Holmes, 2013) and *ggplot2* (v3.5.2) (Wickham, 2011) packages. Statistical comparisons of alpha diversity between healthy and diseased plants were carried out using Wilcoxon rank-sum tests via stat_compare_means() from the *ggpubr* (v0.6.0) package. Different normalization strategies were applied depending on the type of analysis. For beta diversity analyses, count data were normalized using Total Sum Scaling (TSS) (McKnight et al., 2019) and rarefaction was avoided in accordance with best practices (McMurdie and Holmes, 2014). Bray–Curtis dissimilarities were calculated using the *phyloseq* package, and differences in community composition were tested using PERMANOVA with the adonis2() (999 permutations) from the *vegan* package (version 2.6-4) (Oksanen et al., 2001). Group dispersion (i.e., homogeneity of group variances) was evaluated using the betadisper() function from the *vegan* package, which measures the distance of individual samples to their group centroid. This multivariate analog of Levene’s test is commonly used as a complementary approach in beta diversity analysis (Oksanen et al., 2001). Visualization of ordination and test results was performed using *ggplot2* (v3.5.2) (Wickham, 2011). For genus-level analyses, taxonomic aggregation was performed using the tax_glom function from the phyloseq package. Differential abundance analysis was conducted using *DESeq2* v1.49.2 (Love et al., 2014) which models count data using a negative binomial (gamma-poisson) distribution in Deseq(). Count data were normalized using the “poscounts” method, which enables robust estimation of size factors in the presence of a high proportion of zero counts—a typical feature of microbiome data. Result tables including log₂ fold changes, raw *p*-values and adjusted *p*-values (Bonferroni) were extracted and used to generate figures. Differentially abundant taxa were visualized using bar plots in *ggplot2*. Venn diagrams were created using the *ggVennDiagram* package (version 0.1.10) (Gao et al., 2021). All R scripts are available on github at: https://github.com/Leajobert/Rhizosphere_legacy_2025.git.

## 3 Results

### 3.1 Microbiome structure across leaf, root and rhizosphere compartments differs with health status in field-grown rice

Individual plants from the field were selected based on the presence of symptoms on their aerial parts, and DNA was extracted from their leaves, roots, and rhizosphere (six symptomatic plant, referred to as “diseased,” and six asymptomatic plants, referred to as “healthy”). Amplicon sequencing of the V3-V4 region of the 16S rRNA gene and the ITS2 region was then performed to compare their microbiomes. Rarefaction curves (Supplementary Figures S1C–F) confirmed that the sequencing depth was sufficient to capture the bacterial and micro-eukaryotic diversity present in leaves, roots and rhizosphere of field-grown rice. As expected, α-diversity indices of samples followed the gradient rhizosphere > roots > leave for both amplicon markers (Supplementary Figures S1A,B).

**Figure 1:**
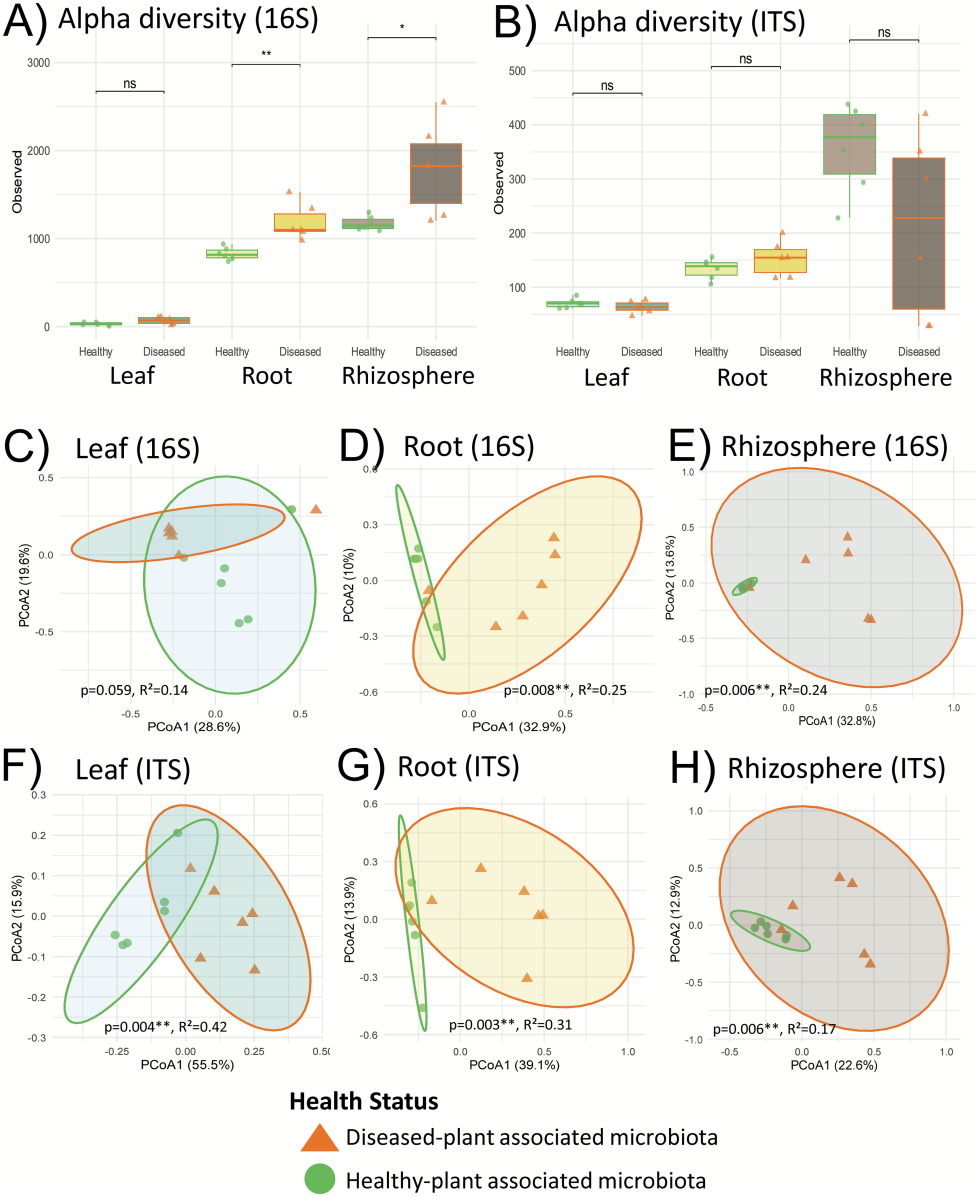
Alpha and beta-diversity analyses of field sample microbiota. **(A,B)** Alpha diversity richness in leaves, roots, and rhizosphere based on 16S **(A)** and ITS2 **(B)** ASV, with statistical comparisons between healthy and symptomatic plants for each compartment. **(C–H)** Beta diversity analysis (PCoA based on Bray–Curtis dissimilarities) showing the effect of health status on microbial communities from the 16S **(C–E)** and ITS **(F–H)** datasets in leaves **(C,F)**, roots **(D,G)**, and rhizosphere **(E,H)**. Statistical tests from **(C-H)** are Permanova tests (999 permutations); *Indicates levels of significativity; *R*^²^ represents the proportion of variation in community composition explained by the Health Status.

For the leaf compartment, α-diversity did not differ significantly between healthy and diseased leaves in either 16S or ITS datasets (Figures 1A,B). β-diversity was also indistinguishable in the 16S data but healthy leaf bacteria microbiota was more dispersed than the one from diseased plants (*p*-value = 0.017*) (Figures 1C,F; Supplementary Table S1). On the contrary ITS profiles separated significantly between the two health states on the PCoA (*p*-value = 0.004**) but dispersion indices did not differ (Supplementary Table S1).

In contrast, root and rhizosphere bacterial communities exhibited clear disease-associated shifts. Both α- and β-diversity differed significantly between healthy and diseased plants for the 16S rRNA gene data (Figures 1A,D,E). For the ITS dataset, α-diversity remained statistically not different between health states in both underground compartments (Figure 1B), but β-diversity showed a marked separation for both below-ground compartments (Figure 1G and Figure 1H). Diseased-plants associated rhizospheres consistently harbored richer and more dispersed microbiota than healthy plants, a pattern highlighted in the PCoA ordinations and calculated in the dispersion indices in Supplementary Table S1.

**Figure 2:**
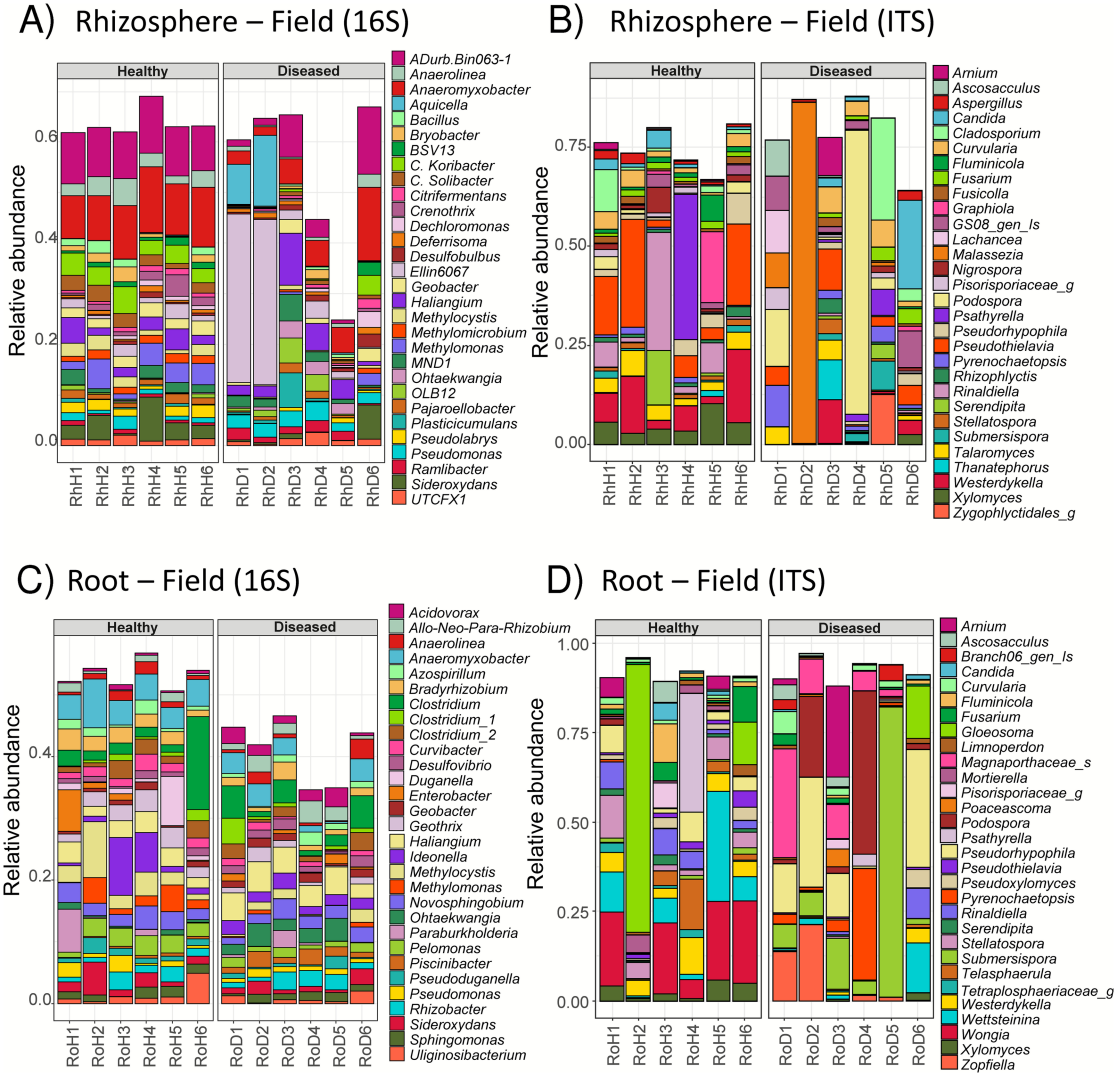
Top 30 microbial genera in the roots and rhizosphere of symptomatic and asymptomatic field rice. Bar plots showing the relative abundance of the top 30 genera in bacterial **(A,C)** and fungal **(B,D)** communities in rhizosphere **(A,B)** and root **(C,D)** samples associated with field-grown rice plants. RhH/RhD, Rhizosphere of Healthy/Diseased Field Plants; RoH/RoD, Root of Healthy/Diseased Field Plants; Is, *Incertae sedis*; g, genus.

Taxonomic binning of the top 30 bacterial and fungal genera from root and rhizosphere samples is presented in Figure 2. Healthy plants exhibit similar profiles in terms of both composition and relative abundance of their top 30 bacterial genera (Figure 2A), a feature also illustrated in the PCoA (Figure 1E). In contrast, diseased plant rhizospheres display more variability: for instance, RhD6 shares a similar top 30 profile with healthy samples, whereas RhD1 and RhD2 are similar to each other but clearly distinct from the others (Figure 2A).

Taxonomic binning of fungal genera shows an even more heterogeneous distribution across individual rhizosphere samples (Figure 2B). Some genera, such as *Xylomyces* and *Westerdykella*, are consistently present in healthy rhizospheres but are largely absent in those from diseased plants. Although RhD1 and RhD2 share similar bacterial profiles, their fungal communities differ markedly: *Malassezia* accounts for over 80% of the fungal relative abundance in RhD2, but only around 10% in RhD1 and is absent in other samples (Figure 2B). In root samples, a similar trend is observed, with highly individualized fungal profiles—often dominated by one or two genera that together make up more than 50% of the total abundance (Figure 2D). In contrast, bacterial genera in roots tend to be shared across samples, albeit at lower relative abundances (Figure 2C). Compared to rhizosphere samples, fewer differences are observed between healthy and diseased plants when examining the top 30 bacterial genera in roots.

### 3.2 Presence and relative abundance of potential bacterial and fungal pathogens in rice leaves

**Figure 3:**
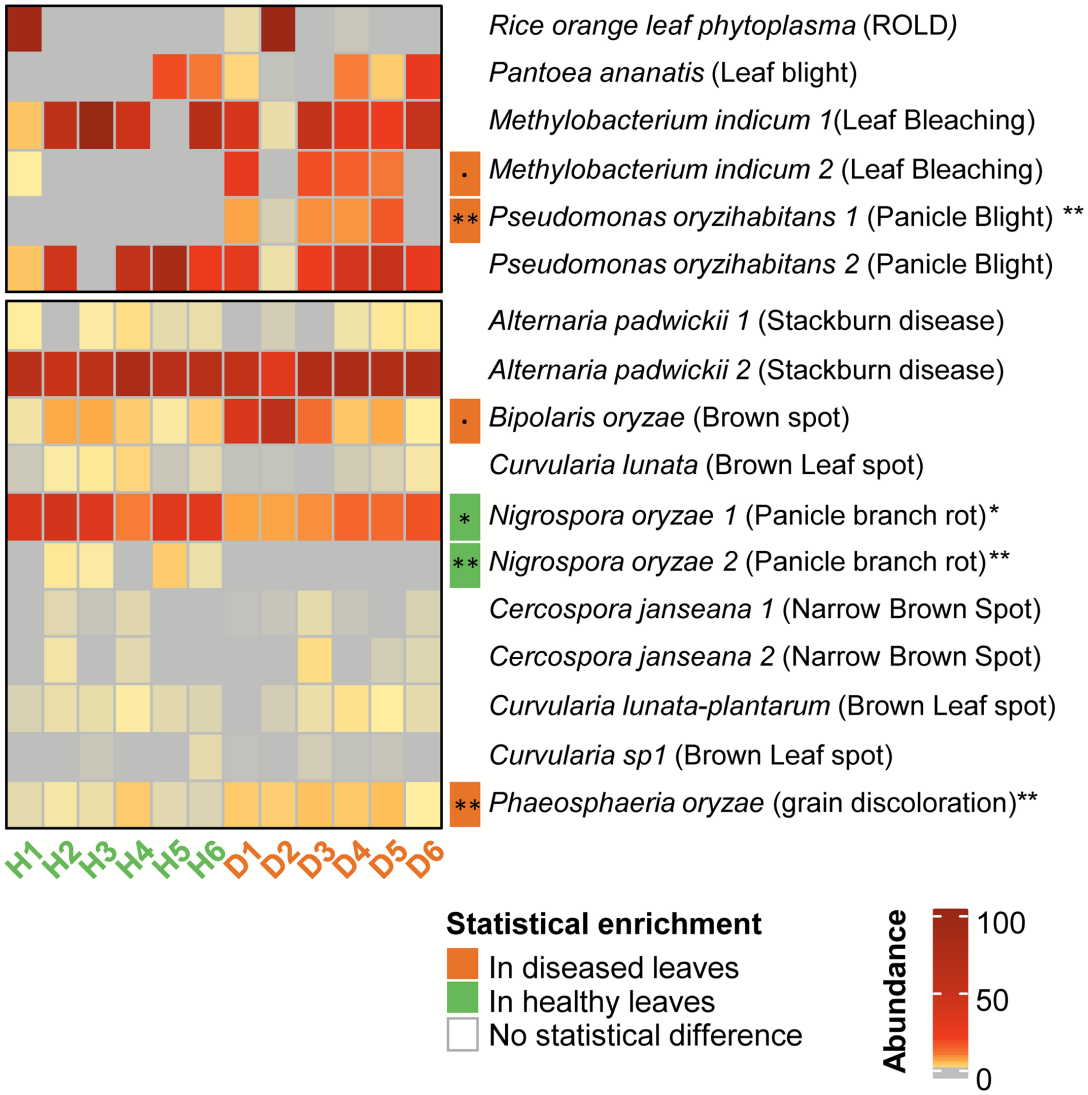
Heatmap of bacterial and fungal pathogens abundance in healthy and diseased rice leaves. This heatmap shows the relative abundance of microbial pathogens detected in 16S and ITS ASV sequences from healthy and diseased leaf samples. The color intensity reflects the relative abundance of each species in each sample. The enrichment columns show a statistical comparison of diseased and healthy samples using a Wilcoxon test, with dot: *p* < 0.1, *p < 0.05, **p < 0.01. Names of phytopathogenic species are numbered when different ASV were affiliated to the same species. Disease name for each pathogen is indicated between brackets.

Figure 3 shows a heatmap of the relative abundance of potential rice-pathogenic taxa in the leaves of the field samples (taxa at species level, only taxa >100 reads across all leaf samples). The complete listing of taxa present in leaves of diseased and healthy plants is shown in Supplementary Figure S2. As the study was initially based on visual assessments of symptoms, this section aims to deepen our understanding of the potential causal agents behind the observed foliar damages. Representative images of leaves from visually classified healthy and diseased plants are provided in Supplementary Figure S3.

Interestingly, a large amount of potential rice pathogens was identified, comprising 6 bacterial species and 11 fungal taxa (Figure 3). Most of these potential rice pathogens are present in both symptomatic and asymptomatic leaves. For example, *Nigrospora oryzae* and *Bipolaris oryzae* are present in all samples, but several pathogens are only present in some plants, as is the case with Rice Orange Leaf Phytoplasma (ROLP) and *Pantoea ananatis*. Statistical analysis revealed some abundance differences between healthy and diseased leaves. *Methylobacterium indicum*2 (*p*-value < 0.1), *Pseudomonas oryzihabitans1* (*p*-value ≤ 0.01) and *Phaeosphaeria oryzae* (*p*-value ≤ 0.01) are enriched in diseased leaves. These pathogens have been reported to cause leaf bleaching, rice panicle blight and grain discoloration, respectively (Lai et al., 2021; Hou et al., 2020). *Nigrospora oryzae*, causal agent of panicle branch rot and leaf spot (Liu et al., 2021) is enriched in healthy leaves but is present in all samples. Examining each plant individually reveals that they all contain taxa known to be pathogenic according to the literature. Additionally, many of the taxa found on the leaves have not yet been studied for their interaction with rice. It cannot be ruled out that some of the taxa presented in Supplementary Figure S2 are also potential rice pathogens.

### 3.3 Composition and differential abundance analysis of rhizosphere microbiota

**Figure 4:**
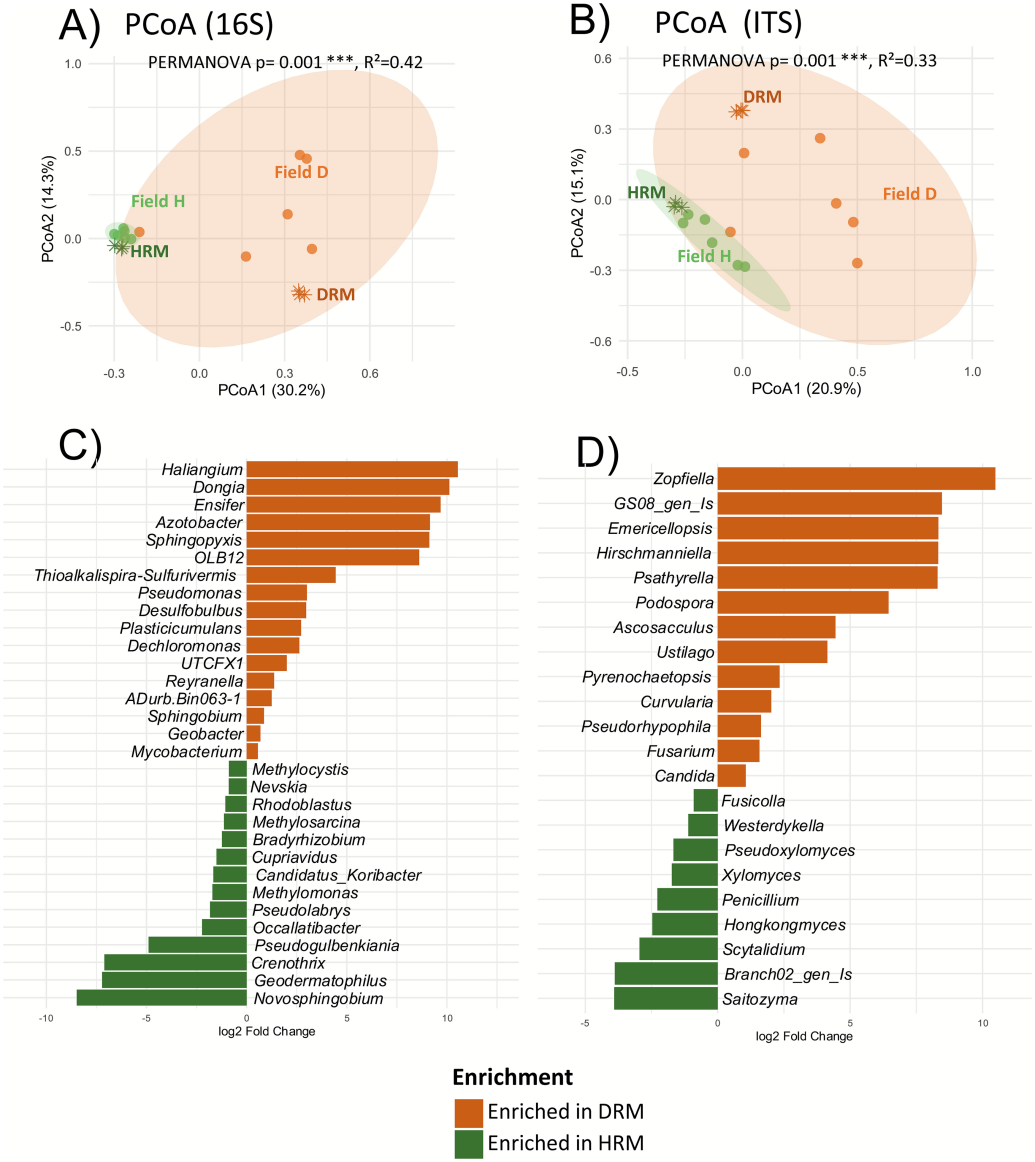
Comparison of microbiota between healthy and disease rhizosphere mix. PCoA plots based on Bray–Curtis dissimilarities of 16S **(A)** and ITS **(B)** datasets of rhizosphere samples collected from field-grown plants [orange dots for diseased plants (Field D), green dots for healthy plants (Field H)], along with the rhizosphere mixes [orange asterisk for disease mix (DRM) and green asterisk for healthy mix (HRM)]. Panels **(C)** (16S) and **(D)** (ITS) display microbial genera that are significantly enriched or depleted between the diseased rhizosphere mix and the healthy rhizosphere mix. Differential abundance analysis was performed using DESeq2, and only genera with log2fold >1 or <1 and adjusted *p*-values (FDR) < 0.05 are shown. Field H/D, Healthy/Diseased Plants from the Field; HRM/DRM, Healthy/Diseased Rhizosphere Mix; Is, *Incertae sedis*; gen, Genus.

Rhizosphere samples from the field were mixed into two groups: the Healthy Rhizosphere Mix (HRM) and the Diseased Rhizosphere Mix (DRM), composed of Healthy (Field H) and Diseased (Field D) plant rhizospheres from the field, respectively. We compared the 16S V3V4 and ITS2 amplicon sequences on each rhizosphere sample as well as their mix. Principal Coordinates Analysis revealed a clear separation of rhizosphere microbial communities based on plant health status, and the microbial profiles of each mix (DRM and HRM) remained closely associated with their respective source communities and very different from each other (Figures 4A,B). HRM samples clustered with Field H, and DRM with Field D, suggesting the stability of these microbial signatures following soil mixing and stabilization. This separation was more pronounced in the bacterial communities, while fungal communities showed a similar but slightly less distinct pattern.

Differential abundance analyses using DESeq2 were performed at genus level between HRM and DRM samples, to identify potential candidates that could explain the observed phenotypes in subsequent generations of plants grown in these two rhizosphere mixes. The differentially abundant genera (padj < 0.05) are represented depending on their log2 fold change in Figure 4C (16S) and Figure 4D (ITS). A total of 17 bacterial genera were significantly enriched in the DRM compared to HRM, while 14 genera were consistently enriched in HRM compared to DRM. Several genera were highly enriched in DRM, such as *Haliangium* (log2fold: x1075), *Dongia* (x800), *Ensifer* (x600) or *Azotobacter* (x410), while other genera were moderately enriched (log2fold < 5), including *Pseudomonas*, *Desulfobulbus, Plasticicumulans* and *Dechloromonas*. In the case of depleted genera in DRM, *Novosphingobium* was the strongest depleted genera (log2fold /850x) followed by *Geodermathophilus, Crenothrix* (/200x), and *Pseudogulbenkiana* (/42x).

Regarding the ITS data, 13 genera were found enriched in DRM compared to HRM, including two fungal genera (*Curvularia* and *Fusarium*) known to host plant phytoparasitic species and one plant root parasitic nematode (*Hirschmanniella*), while nine were depleted. Most of the differentially abundant genera belong to the Ascomycota phylum even though some were Basidiomycota. These results reveal clear microbial shifts in the rhizosphere microbiota of diseased plants versus healthy ones, with enrichment of some genera and depletion of others.

### 3.4 Rhizosphere origin affects plant growth and disease susceptibility

**Figure 5:**
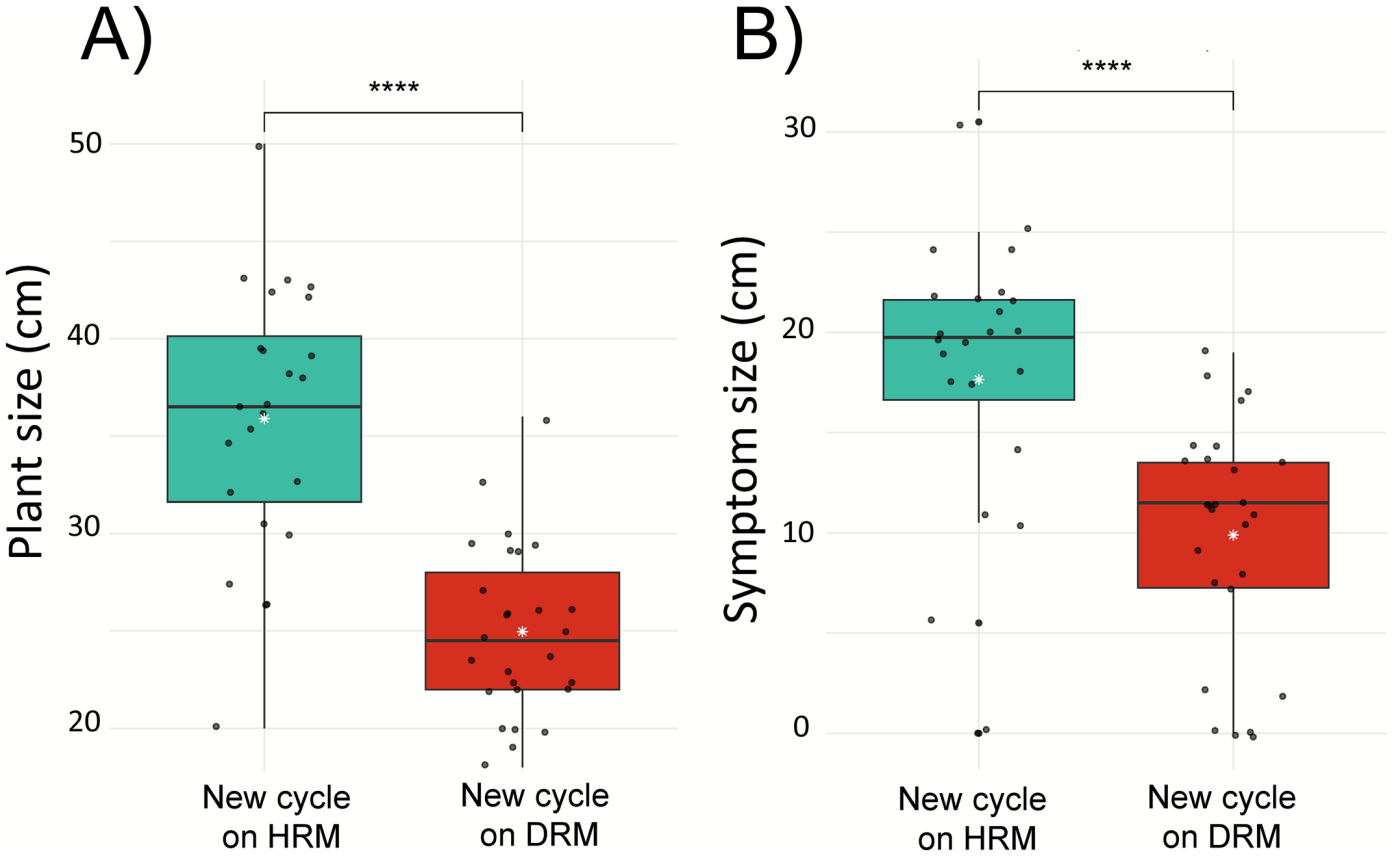
Phenotype of new cycle rice plants grown on rhizosphere mixes derived from healthy or diseased field plants. **(A)** Shoot size comparison of 5 weeks old rice plants grown in HRM or DRM. Wilcoxon test *p* = 4.82e-07 (*n* = 24). **(B)** Lesion length after infection with *Xanthomonas oryzae* pv. *oryzae* (Xoo), depending on the rhizosphere mix in which the plants were cultivated. Wilcoxon test *p* = 7.81e-05 (*n* = 24). HRM/DRM, Healthy/Diseased Rhizosphere Mix. ****Indicates significativity at alpha = 0.01%.

Rice plants (same Srangae Sral variety as in the field) were grown for a new cycle on the DRM and HRM mix under controlled conditions (see Material and Methods), and at 5 weeks of growth, plants were phenotyped for the size of their aerial parts. Plants grown in DRM exhibited significantly reduced shoot growth (*p* < 0.0001) compared to those grown in HRM, corresponding to a 30% decrease in shoot length (Figure 5A). Two weeks after leaf clipping, the length of plant symptoms induced by Xoo were measured. Interestingly, despite their smaller size, plants grown on DRM developed significantly shorter leaf lesions (*p* < 0.0001) following Xoo infection corresponding to a 44% symptom length reduction in the new cycle plants grown on DRM compared to those grown on HRM (Figure 5B).

### 3.5 Rhizosphere microbiota structure and transmission across plant generations

**Figure 6:**
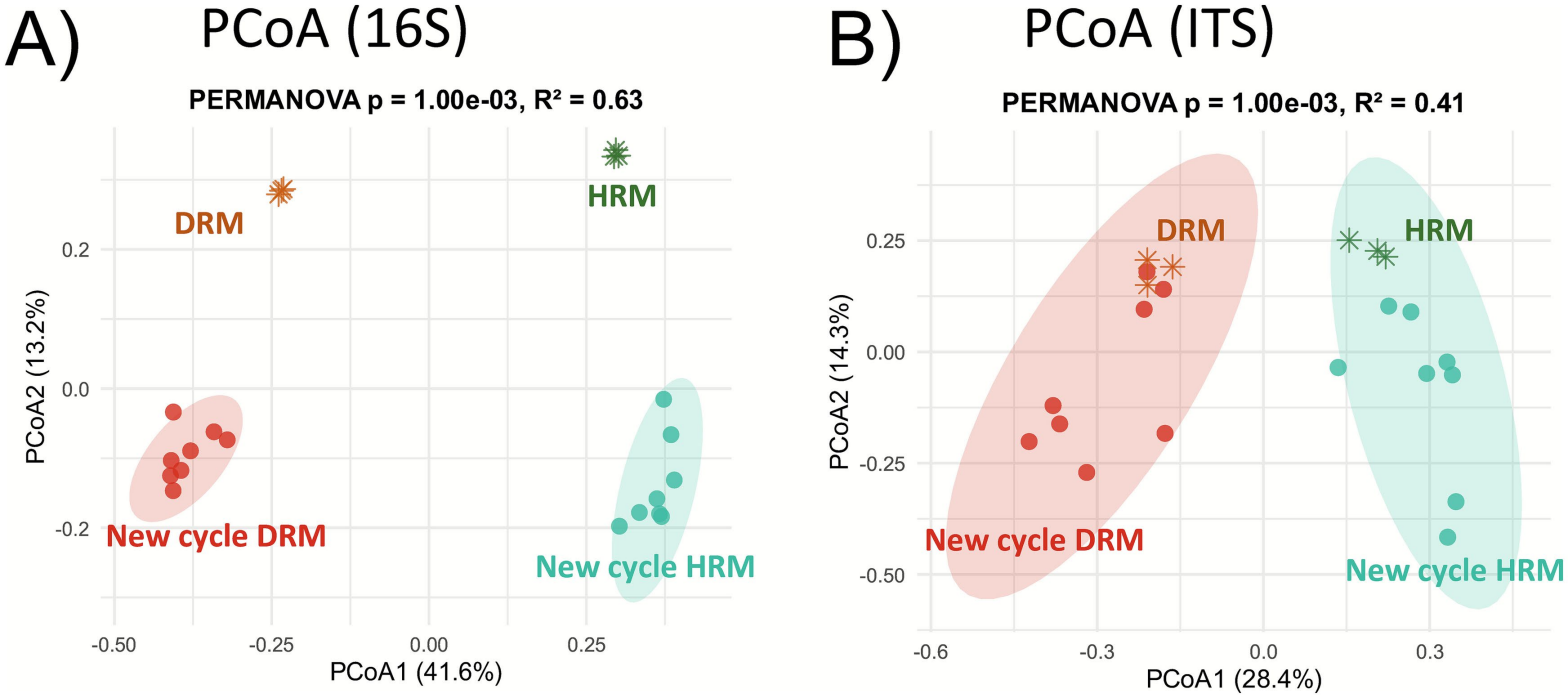
Comparison of the microbiome between the rhizosphere mix and the rhizosphere of the next plant generation. PCoA plots based on Bray–Curtis dissimilarities of **(A)** 16S and **(B)** ITS datasets of the DRM (Disease Rhizosphere Mix) and HRM (Healthy Rhizosphere Mix) and from the rhizosphere of new plant generation (New cycle DRM/New cycle HRM).

To evaluate the structure and legacy transmission of rhizosphere microbial communities, we compared the bacterial and eukaryotic communities in the field rhizosphere mix (DRM and HRM) and in the rhizosphere of the new cycle of plants grown on these mix (New cycle DRM/HRM). Principal Coordinates Analysis (PCoA) was performed based on Bray–Curtis dissimilarities and is presented in Figure 6. In the bacterial dataset (Figure 6A), the DRM and HRM formed clearly separated clusters along the first principal coordinate (PCoA1, 41.6%). The rhizosphere bacteria of the new cycle plants also formed distinct groups, separated from the original mixes along the second principal coordinate (PCoA2, 13.2%). The bacterial communities of new cycle plants clustered according to their respective inoculum sources. The same pattern was observed on fungal communities except that new cycle plants did not impact global community structure compared to the rhizosphere mix as much as they did for bacteria. Nonetheless, rhizosphere bacterial and fungal communities from plants grown in DRM remained clearly separated from those grown in HRM, and new cycle plant rhizospheres did not seem to cluster closer together with this new cycle in controlled conditions. Together, these results underscore the lasting impact of disease-associated rhizosphere microbiota on shaping microbial communities in the next plant generation.

### 3.6 Inherited genera across generations and differences depending on health status of the field plants

**Figure 7:**
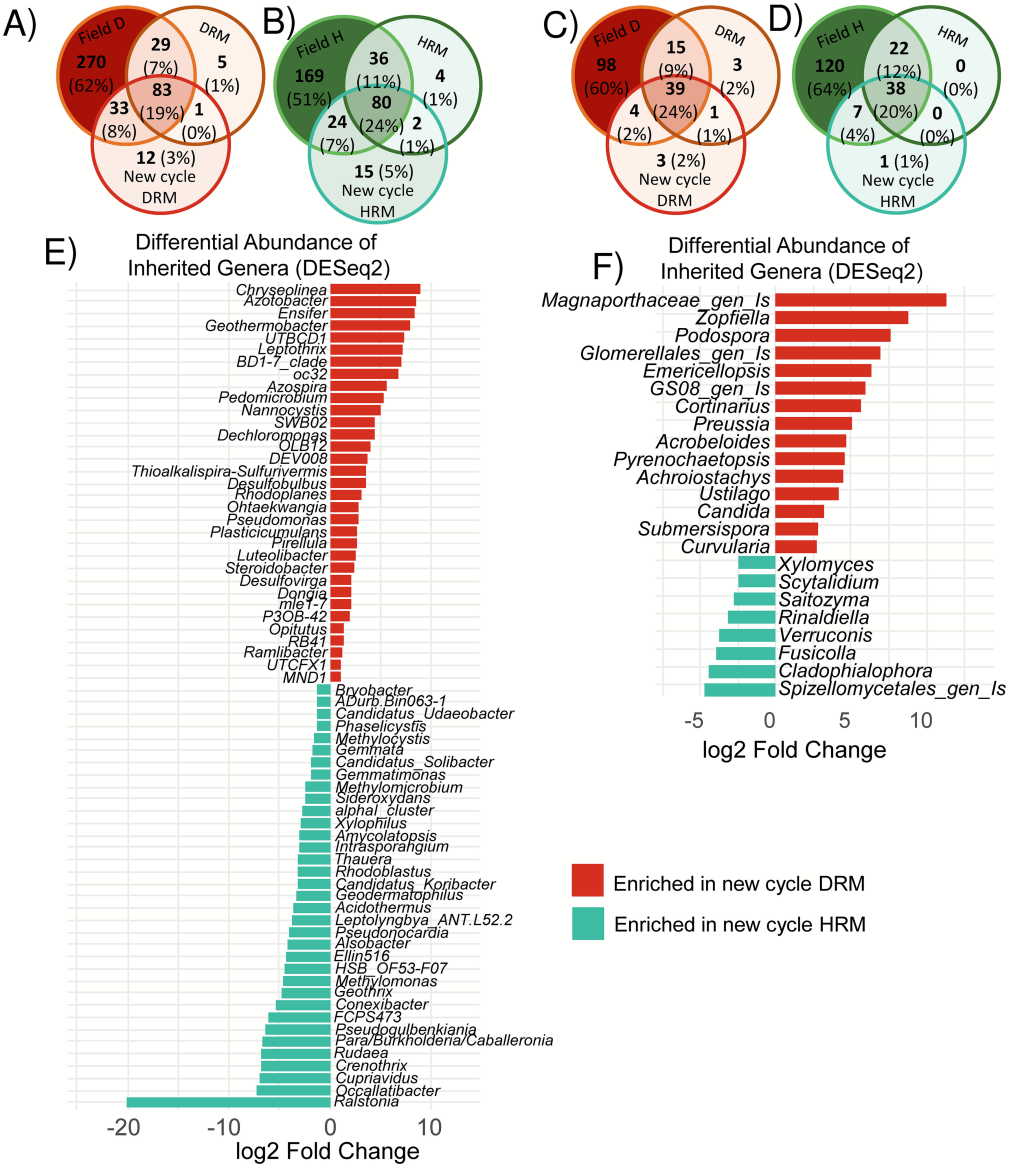
Transmission of rhizosphere microbial taxa and differential abundance analysis. Panels **(A–D)** show Venn diagrams illustrating the overlap of genera found in the rhizospheres of field-grown plants, the rhizosphere mixes, and the rhizospheres of next-generation of plants. Data are shown for 16S in diseased **(A)** and healthy **(B)** conditions, and for ITS in diseased **(C)** and healthy **(D)** conditions. Genera shared across all three groups were used for a differential abundance analysis using DESeq2: **(E)** for 16S and **(F)** for ITS. Bar plots display genera significantly enriched in the rhizosphere of next-generation plants (adjusted *p*-value (FDR) < 0.05). HRM/DRM, Healthy/Diseased Rhizosphere Mix.

Venn diagrams summarizing bacterial (16S) and microeukaryotic (ITS) datasets were used to track genera present in rhizospheres from the field, in the stabilized rhizosphere mixes, and in the rhizospheres of the new cycle plants (Figures 7A–D). A genus was considered inherited if it occurred in at least 50% of samples of the new cycle plants rhizosphere, whereas no prevalence filter was applied to field samples because of their high compositional heterogeneity due to the experimental design. Among bacteria, 19% of genera in the diseased series and 24% in the healthy series were transmitted across all three stages; the corresponding values for microeukaryotes were 24 and 20%. The majority of genera were only found in field plants, representing 62–51% of the 16S dataset and 64–60% of the ITS dataset. Genera shared exclusively by field samples and the rhizosphere mix accounted for 7–12%, while those detected only in new cycle plants constituted 1–5%, and a small subset of genera appeared in field and new cycle samples but not in the mix.

Differential abundance analysis of the inherited genera (Figures 7E,F) identified 33 bacterial genera enriched in the diseased legacy (of which 30% were already enriched in the mix, Figure 4C) and 35 enriched in the healthy legacy (padj < 0.05). In the microeukaryotic core, 15 genera were enriched in the diseased legacy (of which 53% were already enriched in the mix, Figure 4D) and eight depleted in the healthy legacy. Most of the differentially abundant genera were already identified in the rhizosphere mix comparison (Figures 4C,D). The most enriched bacterial taxa in the diseased legacy were *Chryseolinea*, *Azotobacter* and *Ensifer*, and for fungal genera a new genus of the *Magnaporthaceae* family, *Zopfiella* and *Podospora*. No bacterial or fungal genera known to host rice phytopathogens could be spotted in the enriched diseased legacy, with the exception of the bacterial *Pseudomonas* genus (that has *P. fuscovaginae* and *P. oryzihabitans* described as rice pathogens) and the fungal *Curvularia* genus hosting the rice leaf phytopathogen *Curvularia lunata.* For the healthy legacy, the most enriched genera were *Ralstonia*, *Occallatibacter* and *Cupriavidus* for bacteria, and a genus of the *Spizellomycetales* order*, Cladophialophora* and *Fusicolla* for fungi.

## 4 Discussion

Plants have coevolved with complex microbial communities, shaping their interactions with both beneficial and pathogenic organisms. Among these, the rhizosphere microbiome—inhabiting the narrow soil zone surrounding roots—plays a central role in modulating plant health, influencing nutrient uptake, stress tolerance, and disease outcomes (Bakker et al., 2020). While the importance of the rhizosphere in plant–pathogen dynamics is increasingly recognized, relatively few studies have examined how rhizosphere soil microbial communities differ between symptomatic and asymptomatic plants growing side by side within the same field, or what mechanisms underlie these differences (Dastogeer et al., 2022). Moreover, the microbial legacy of a plant can persist beyond its lifetime and impact next generation resistance as shown through experimental evolution experiments (Yuan et al., 2018).

In our study we explored whether the concept of diseased-induced modifications of the plant microbiome occurs in crops grown in an agronomic soil and if its legacy has an impact on the next generation. We have evidenced that the root-associated microbiome of rice plants in the field is deeply impacted by the presence of symptoms on leaves. Furthermore, transferring the rhizosphere of such plants to a new plant cycle limited plant growth and decreased symptoms caused by Xoo infection. The comparison of the microbiota composition of the diseased rhizosphere mix (or new cycle plants grown on it) and the healthy rhizosphere mix (or new cycle plants grown on it) did not identify strong rice phytopathogens (either soil-borne or foliar) that could explain this phenotype. This suggests that there is a microbiome imbalance (or dysbiosis) in plants grown on the DRM as a result of biotic stress from the previous cycle.

### 4.1 Rhizosphere and root dysbiosis parallels foliar symptoms

Dysbiosis, usually defined as an imbalance in hosts microbiota implying a reduction of holobionts fitness, is an ambiguous concept. It can be either cause and consequence of stress or disease which is why the concept has been criticized (Olesen and Alm, 2016). Characteristics associated with it are not always clear, even though often associated with a change in α-diversity and increase of β-diversity (Arnault et al., 2023). In our study, based on the phenotype of the plant collected in the field (healthy vs. diseased), we detected an increase of β-diversity and more variability among diseased plants than healthy in the rhizosphere and root microbiomes of plants. The diseased plant-associated microbiota also display more variability among themselves than healthy ones, a feature well visualized when looking at the top 30 bacterial genera associated with the rhizosphere of healthy plants in comparison to diseased one. This feature fits with the Anna Karenina Principle that was proposed to be applied to plant microbiomes in the Arnault et al. (2023) review, “All healthy microbiota are alike; each disease-associated microbiota is sick in its own way” (Arnault et al., 2023). Similar increases in bacterial β-dispersion of diseased plant microbiomes have been documented, for example during disease progression in tomato infected by *Ralstonia solanacearum* (Wei et al., 2018), and are generally interpreted as ecological destabilization. A meta-analysis (the Microbiome Stress Project) found that exposure to stressors increases the beta diversity of the microbiome globally, across a range of environments and types of stressor (Rocca et al., 2019). One important point to assess, is that our dataset only comprises one timepoint and thus cannot resolve whether dysbiosis is a cause or consequence of foliar disease. If we assume that the leaf symptoms are a consequence of dysbiosis, then this implies that the distribution of diseased plants in the field is itself the result of an inheritance of the rhizosphere microbiomes from the previous generation. Since sampling roots and the rhizosphere is destructive, it is difficult to carry out temporal monitoring in the field that would allow us to decide between the two hypotheses. One study managed to monitor the microbiome composition of tomato from seedlings to infected plants, emphasizing that initial microbiomes preprogram future plant health (Wei et al., 2019).

### 4.2 Phytopathogens occurrence does not explain leaves symptoms

As our study relied on the observation of symptoms on rice plant leaves in the field, we attempted to identify the leaf pathogens that were potentially responsible for these symptoms. Microbiome analysis of the leaves of healthy and diseased plants revealed a diverse range of bacterial and fungal taxa. We detected two bacterial species significantly enriched in the leaf microbiome of symptomatic plants (*Pseudomonas oryzihabitans* and *Methylobacterium indicum*), but if these two species contain strains described as potential rice pathogens (Lai et al., 2021; Hou et al., 2020), there is very few studies describing their pathogenicity, and *M. indicum* was found as a strong health-signature taxa in the leaves of rice in another study (Oeum et al., 2024). On the fungal side, two phytopathogens were detected as significantly enriched in symptomatic leaves, including *Bipolaris oryzae*, responsible for brown spot (Savary et al., 2019), and *Phaeosphaeria oryzae*, a fungal species suspected to be involved in rice grain discoloration but without clear demonstration. Other phytopathogenic species were detected, including *Alternaria padwickii* (stackburn disease), *Cercospora janseana* (narrow brown spot) and *Curvularia lunata* (brown leaf spot), but there was no difference in their prevalence between symptomatic and asymptomatic leaves.

Overall, “healthy” plants did not exhibit a lower frequency of potential rice phytopathogens than diseased plants. There was also no clear pattern of pathogens that could explain the symptoms observed in individual plants (except for *Bipolaris oryzae*, which was enriched in several symptomatic plants but was also present in asymptomatic ones). As we did not monitor viruses, we cannot rule out the possibility that this type of pathogen could also explain some of the symptoms. Further work using culture-based isolation and pathogenic tests would be needed to identify the causing agents. The pathogen distribution being not a clear explanation of disease occurrence (healthy and symptomatic leaves carrying phytopathogens), other hypotheses linked to microbiome composition could explain the healthy state, as we observed differences in microbiome composition in the leaves, roots and rhizosphere. There is undoubtedly a subtle balance between the structure of the rhizosphere microbiome, the density of pathogenic bacteria on leaves, and the development of disease. If we could not determine clearly a single origin and cause of disease of our studied plants from the field, what we observe is a strong correlation between the presence of symptoms and modifications in the plant microbiome, including its rhizosphere.

### 4.3 The rhizosphere legacy shapes the growth and defense of the next generation of plants

Transplanting the two contrasting rhizospheres (DRM/HRM) into a new rice generation revealed a clear legacy effect. Plants grown in the “diseased” mix were significantly smaller than the plants grown on the “healthy” mix, yet significantly less susceptible to *Xanthomonas oryzae* pv*. oryzae*, a rice pathogen often recovered from Cambodian rice fields (Oeum et al., 2024) but undetected in our field plants. There is therefore a dual effect of the soil of origin on the plant, in terms of growth and resistance to pathogens. Various explanations can be suggested to explain this result. The microbiota associated with diseased field plants may prime host immunity or modulate defense signaling pathways, thereby enhancing resistance to foliar pathogens in the subsequent generation under controlled conditions. Similar improvements in defense have been reported in *Arabidopsis*, where downy mildew infection recruits a protective microbial consortium that suppresses subsequent pathogen development (Goossens et al., 2023) and in tomato, where rhizosphere transfer from resistant to susceptible cultivars conveys resistance to *R. solanacearum* (Kwak et al., 2018). In terms of plant growth, most studies describing enhanced defenses in pathogen-influenced soils do not report the growth performance of the new generation, making our results difficult to compare directly. One study in maize showed a similar pattern, with reduced growth and increased defense, in one of the two tested genotypes (Hu et al., 2018).

One alternative explanation to observed phenotypes is that the DRM inoculum contains plant pathogens and/or, led to a stress response in plants and activation of defense pathways—a response consistent with the so-called growth–defense trade-off (He et al., 2022). Indeed, in plants the activation of defense mechanisms often comes at the expense of growth and development, reflecting a well-documented trade-off between these two essential biological processes (Karasov et al., 2017). While this trade-off enables plants to prioritize survival under biotic stress, it can limit biomass accumulation. The adverse effects of inoculated plant growth-promoting bacteria (PGPB) on plant growth are numerous. Certain PGPB strains produce bioactive metabolites, such as antibiotics, volatile organic compounds or excessive phytohormones, that can shift from beneficial to detrimental depending on the environmental context (Etesami, 2025).

An overall unbalanced microbial community may also strongly impact plant growth. The initial DRM mix itself comes from rhizosphere soils that are very different from one another. The combination of these soils may have generated a very atypical, unbalanced community, which could therefore have a negative impact on the plant in terms of growth but, conversely, stimulate its immunity through this imbalance, resulting in better resistance to pathogen inoculation. These findings raise important questions about the functional consequences of microbial legacy effects. Although the diseased rhizosphere mix (DRM) negatively impacted plant growth, it also appeared to prime a stronger immune response, as reflected in the lower susceptibility to *X. oryzae* pv. *oryzae*. The microbial community inherited from the DRM was enriched in genera previously associated with pathogenicity or stress-related functions, such as fungal phytopathogens (*Curvularia lunata, Fusarium* spp.) or root phytoparasitic nematodes (*Hirschmanniella* spp.). This supports the hypothesis that exposure to a stress-associated microbiota can lead to an early or constitutive activation of plant defenses—at the expense of growth. Future work should explore this growth–defense trade-off more directly, for example through transcriptomic analyses to test the induction/priming of plant defense genes combined with hormone profiling. Furthermore, in terms of fitness, it would be advisable to measure the impact this may ultimately have on the number, weight and quality of the grains produced.

Repeating the experiment with other pathogens and in different field settings would also help to assess the generality of the disease-induced microbiome response. Such approaches align with growing evidence that positive plant-soil feedbacks, driven by beneficial microbes persisting across generations, can be leveraged for sustainable crop protection (Spooren et al., 2024), but that negative feedbacks can also play a major role, for example by having a detrimental effect on plant growth. Indeed, the soil legacy inherited from plant-soil feedbacks can go both ways (Van der Putten et al., 2013).

### 4.4 Microbiota sampling and microbial taxa transmission across generation

Only a limited number (19 to 24%) of microbial genera, either bacterial or fungi, were consistently transmitted across the three stages (field, mix, and greenhouse). A large proportion of the microbial taxa were only present in the initial rhizosphere soils, and absent from the mix and from the next cycle plants. So, there was a strong bottleneck in the transmission from the field to the next generation test, that may be due to the great heterogeneity of the soils that were mixed, and/or to the effect of the three-week stabilization period for the mix that may have lowered the diversity. A decrease in soil bacterial richness and a reduction of within-group heterogeneity following soil mixing has already been reported (West and Whitman, 2022). In contrast, Huet et al. (2025) found no differences in alpha-diversity, but mainly shifts in the composition of the resulting coalesced communities. More broadly, there is a recognized lack of empirical research into how coalescence affects drift and diversification, and how these processes shape community outcomes (Liu and Salles, 2024). We therefore acknowledge that our mixed soils only partially reflect the starting communities, which raises the question of the relevance of soil mixing in this type of experiment. Nevertheless, despite the loss of a proportion of field taxa during this transition, the inherited microbiota remained clearly distinct between HRM and DRM, supporting the robustness of the observed legacy effect. Genera found exclusively in the rhizosphere of new cycle plants constituted 1–5% of all genera, probably reflecting low-level environmental contamination from water or air contaminants, or falling below the sequencing detection threshold. A small subset of genera appeared in field and new cycle samples but not in the mix, suggesting their abundance in the mix fell below the detection threshold of the amplicon sequencing. The sequencing depth of amplicon sequencing (50,000 reads per library in our study) might thus be increased to allow detecting low frequency taxa and better follow their evolution across generations.

## 5 Conclusion

Using an initial sampling in the field, based on plants with or without symptoms of disease, our study shows that the effects of this state of health are passed on to the next generation via the rhizosphere and its composition. We have carried out this study at the fine scale of the plant and its root system. We found that while a healthy plant (i.e., without symptoms but with the potential presence of pathogenic microorganisms) structures its microbiome homogeneously, diseased plants (with symptoms) are characterized by an unbalanced root-associated microbiome that varies greatly from one individual to another. This result reinforces the idea that a healthy plant relies on a microbiome whose composition is adapted and regulated. We have carried out this experiment on a single cycle only. It would be interesting to see the dynamic evolution and restructuring of the microbiome over successive cycles, with or without foliar pathogen pressure. Together, these results underline the potential for manipulating soil microbiomes to modulate crop performance and disease outcomes, applying approaches to promote a microbiome as close as possible to that of a healthy plant. We show, however, the complexity of transferring a microbiome from one generation to the next, with massive losses of taxa, due in part to random bottleneck effects. The ideas of microbiome transposition, by inoculation with soil from another site, therefore appear complex to implement. It would also be interesting to see to what extent, in a field, at an individual plant level, the effects we are observing (reduced growth, better resistance to a pathogen) are perpetuated from one generation to the next, with or without tillage, which would modify the soil structure.

## Data Availability

The datasets presented in this study can be found in online repositories. The names of the repository/repositories and accession number(s) can be found below: https://www.ebi.ac.uk/ena, PRJEB84191.

## Footnotes

1
https://www.ebi.ac.uk/ena

2
https://blast.ncbi.nlm.nih.gov/Blast.cgi
