# Supplementary material for: Rhizosphere legacy of leaf-diseased rice and its impact on next generation
Source: Front Microbiol. 2025 Dec 17;16:1677271. doi: 10.3389/fmicb.2025.1677271 (PMC12753873; doi:10.3389/fmicb.2025.1677271)
Supplement: Supplementary file 1 [file Table_1.pdf]

Supplementary Table S1: Beta-diversity and dispersion of field-grown plants

Complementary data to figure 1. (A) 16S rRNA of (B) ITS associated dispersion of beta-diversity of Healthy and Diseased plants for each compartment and statistical test

A) 16S

Compartment	Dispersion Healthy	Dispersion Diseased	P-value
Leaf	0.5707	0.3451	0.017*
Root	0.4280	0.4596	0.505
Rhizosphere	0.3895	0.5616	0.001 ***

B) ITS

Compartment	Dispersion Healthy	Dispersion Diseased	P-value
Leaf	0.1637	0.1740	0.786
Root	0.3838	0.4540	0.406
Rhizosphere	0.6121	0.4484	0.01**
