# Supplementary material for: Rhizosphere legacy of leaf-diseased rice and its impact on next generation
Source: Front Microbiol. 2025 Dec 17;16:1677271. doi: 10.3389/fmicb.2025.1677271 (PMC12753873; doi:10.3389/fmicb.2025.1677271)
Supplement: Supplementary file 2 [file Data_Sheet_1.pdf]

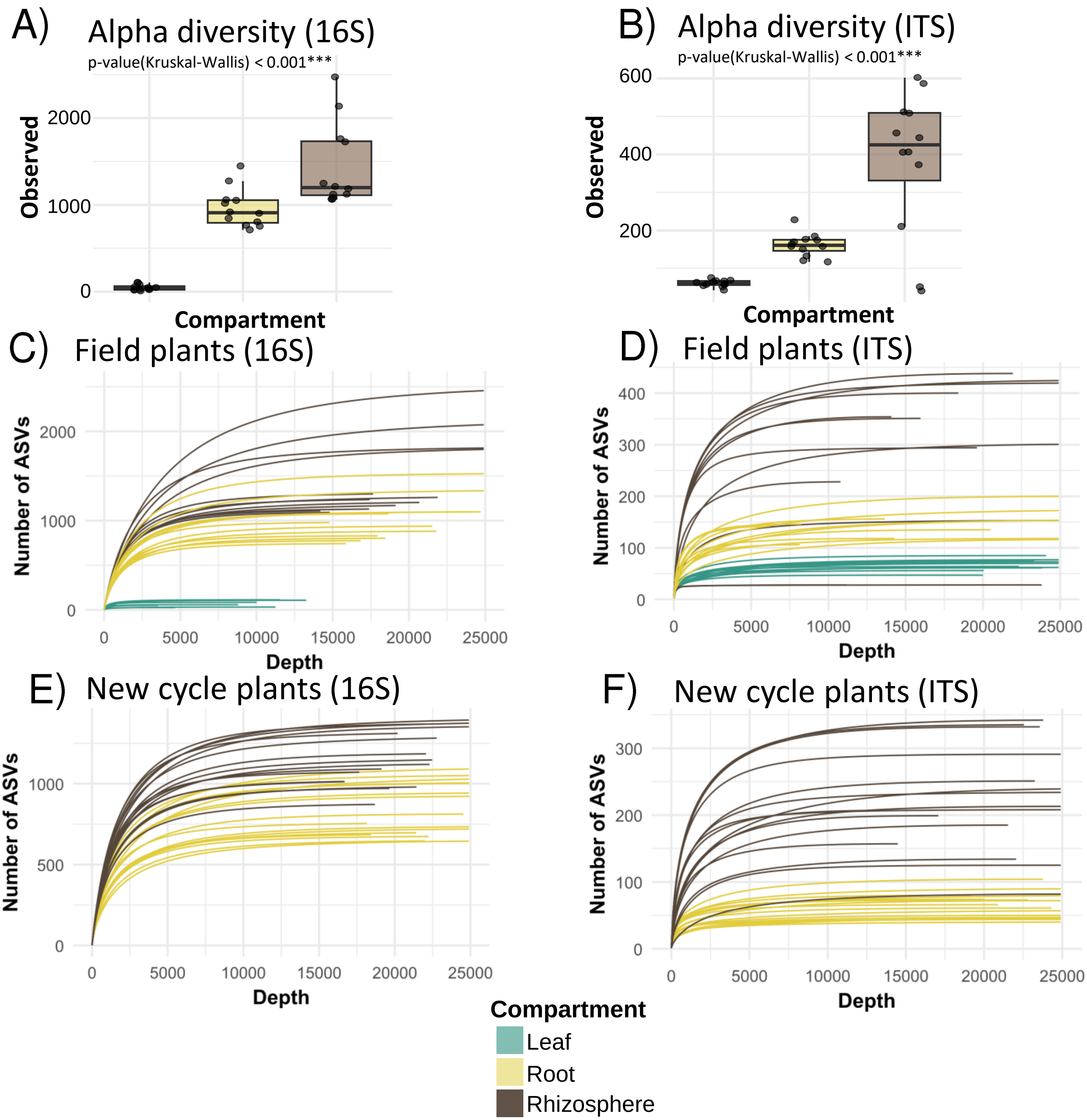

Supplementary Figure S1: Alpha diversity and rarefaction curves

Alpha diversity comparison between compartments (leaves, roots and rhizosphere) in field-grown plants for (A) the 16S rRNA dataset and (B) the ITS dataset.

Rarefaction curves and microbial richness are shown for: (C and D) microbiota associated with field-grown plants based on 16S (C) and ITS (D) sequencing; (E and F) microbiota associated with next-generation plants grown under controlled conditions based on 16S (E) and ITS (F) sequencing.
