# Supplementary material for: Rhizosphere legacy of leaf-diseased rice and its impact on next generation
Source: Front Microbiol. 2025 Dec 17;16:1677271. doi: 10.3389/fmicb.2025.1677271 (PMC12753873; doi:10.3389/fmicb.2025.1677271)
Supplement: Supplementary file 3 [file Data_Sheet_2.pdf]

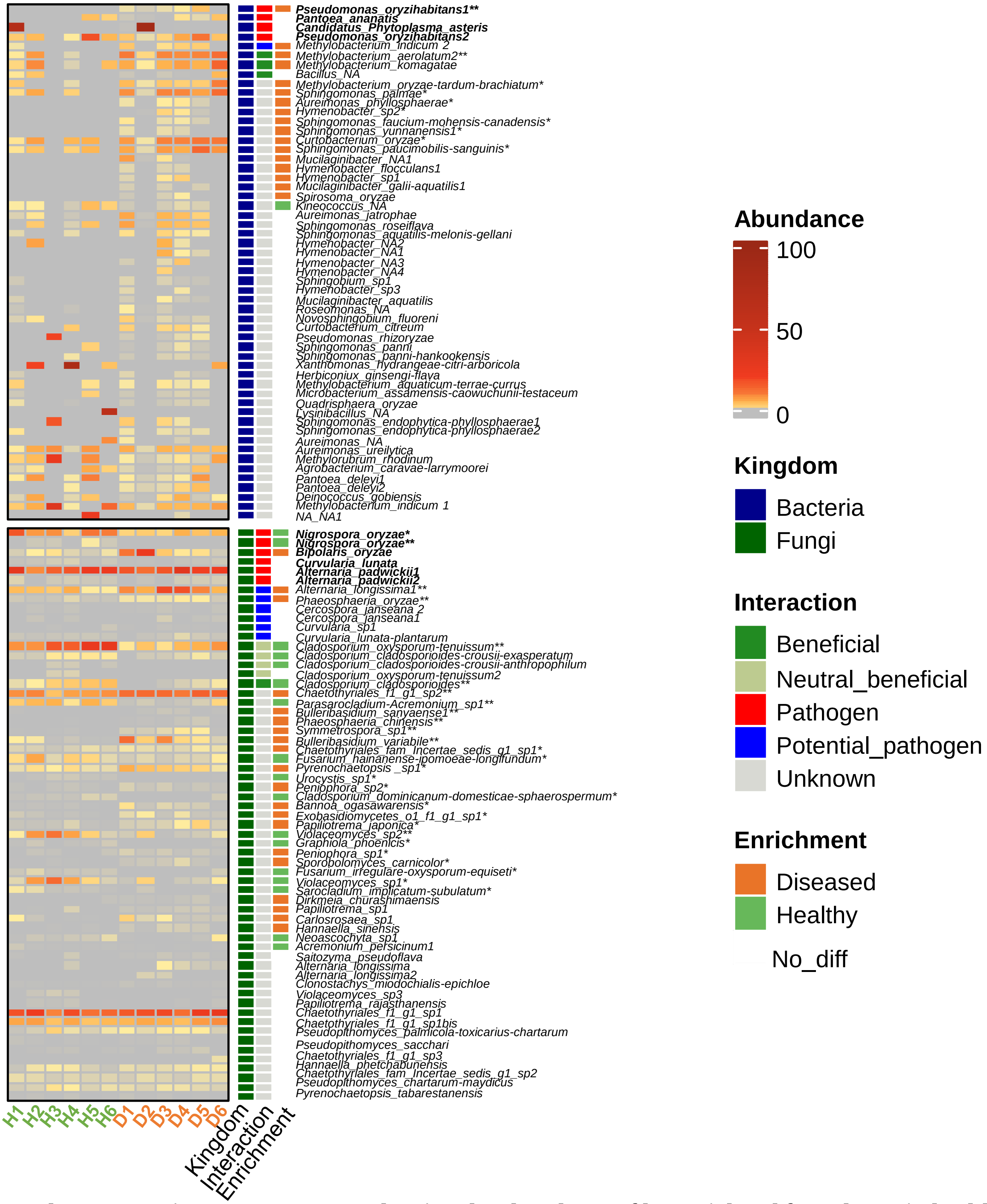

Supplementary Figure S2: Heatmap showing the abundance of bacterial and fungal taxa in healthy and diseased rice leaves.

This heatmap illustrates the relative abundance of all microbial taxa identified in the 16S and ITS amplicon sequence variants (ASVs) from healthy and diseased leaf samples. The colour intensity reflects the abundance of each species in each sample. The first column shows bacterial taxa in blue and fungal taxa in green. The second column represents the putative interaction of these microorganisms with rice, as described in the literature. The third column, representing enrichment, shows a statistical comparison of diseased and healthy samples using a Wilcoxon test: with dot: p<0.1, *:p<0.05,**:p<0.01. The names of phytopathogenic species are numbered when different ASVs are affiliated with the same species. Abbreviations: o = order; fam/f = family; g = genus; sp = species.
