# Supplementary material for: Rhizosphere legacy of leaf-diseased rice and its impact on next generation
Source: Front Microbiol. 2025 Dec 17;16:1677271. doi: 10.3389/fmicb.2025.1677271 (PMC12753873; doi:10.3389/fmicb.2025.1677271)
Supplement: Supplementary file 4 [file Data_Sheet_3.pdf]

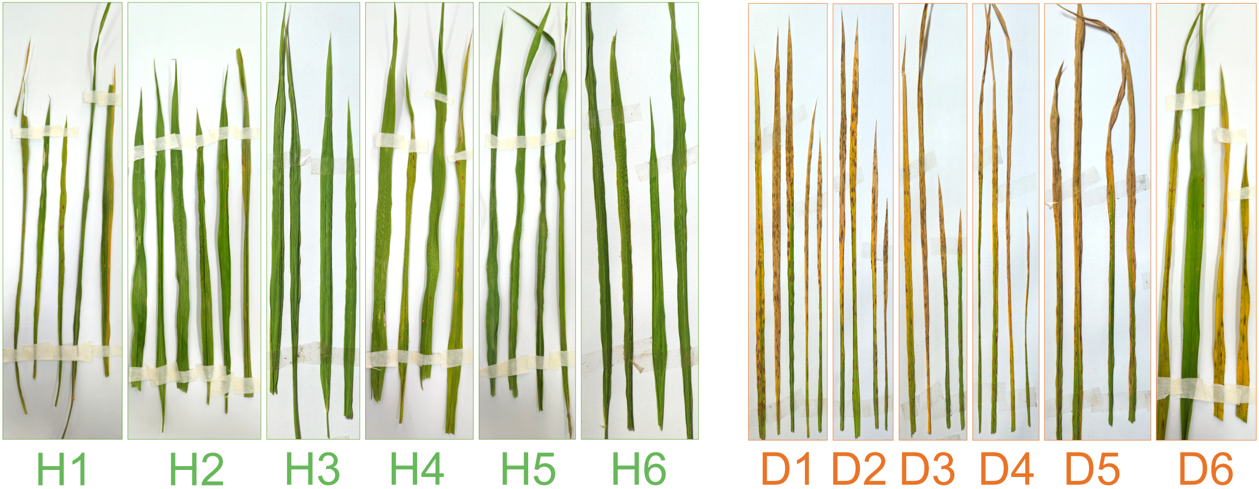

Supplementary Figure S3: Pictures of rice leaves sampled in the field.
Photographs of symptomatic (D1–D6) and asymptomatic (H1–H6) rice leaves collected from the field, selected based on the presence or absence of visible disease symptoms.
